# Evaluation of a community-based randomized controlled prenatal care trial in rural China

**DOI:** 10.1186/1472-6963-11-92

**Published:** 2011-05-04

**Authors:** Zhuochun Wu, Kirsi Viisainen, Ying Wang, Elina Hemminki

**Affiliations:** 1 School of Public Health, Fudan University, 138 Yi Xue Yuan Road, Shanghai 200032, China; 2 Department of Public Health, University of Helsinki, Helsinki, Finland; 3 THL, National Institute for Health and Welfare, Helsinki, Finland

## Abstract

**Background:**

A community-based randomized control prenatal care trial was performed in a rural county of China during 2000-2003. The purpose of this paper is to describe the trial implementation and the impact of the trial on the utilization of prenatal care and perinatal outcomes.

**Materials and methods:**

In the study county, 10 townships (from a total of 55) were each paired with a control (20 study townships in total), with the criteria for pairing being the township's socioeconomic development, perinatal health, and maternal care utilization and provision. One of each township pair was randomly allocated to the intervention or control groups. The trial interventions were: 1) training township hospital midwives and instructing them in how to provide systematic maternal care, 2) informing women in the community of the importance of prenatal care, 3) if needed, providing basic medical instruments to the hospitals. A variety of data sources were used to describe the trial implementation (observations, group discussions, field notes, survey to women). The data on pregnancy and perinatal outcomes were from the original hand-written work-records in the village family planning centers of the study townships.

**Results:**

Implementation of the intervention was deficient. The factors hindering the trial implementation included poor coordination between midwives and family planning officers, broader policy changes implemented by the provincial government during the trial, the decentralization of county governance, and the lack of government funding for maternal care. There was only little difference in the use of maternal care, in women's opinions related to maternal care or content of prenatal care, and no difference in the perinatal outcomes between the intervention and control townships.

**Conclusions:**

A community based randomized controlled trial could not be fully carried out in rural China as planned due to the changing political landscape, the complexity of the socio-economic situation and a lengthy planning stage. The study could not answer if perinatal outcomes could be improved by increased use of prenatal care.

**Trial registration:**

NCT 01054235

## Background

Prenatal care has become routine care for pregnant women in the developed world, and increasingly in developing countries too [1-4]. Prenatal care is assumed to improve maternal and neonatal health notably, though this has not been demonstrated conclusively in studies [5-10]. The wide variation in the design and implementation of prenatal programs has made it difficult to determine the effectiveness of prenatal care [11]. Another major challenge in evaluating prenatal care is adequately controlling confounders and selection bias, with some studies trying to achieve this with the use of quasi-experimental evaluation designs. Only a few studies have adequately controlled for socioeconomic status, which is a composite variable of occupation, education and income [5,12,13].

The Chinese government has promoted nationwide prenatal care since 1987. The Ministry of Health issued guidelines entitled "Perinatal Care Management Approaches for Urban Women" and "Systematic Maternal Care Management Approaches for Rural Women" in 1987 and 1989, respectively [14]. Since then, both the provision and utilization of prenatal care have increased, although discrepancies continue both between urban and rural areas and within them. Especially in rural areas, prenatal care varies from place to place in terms of the timing, frequency and the content of prenatal care [15]. By the late 1990s a few rural areas still had not organized systematic prenatal care. This situation offered the possibility to conduct a randomized controlled trial to evaluate the effectiveness of prenatal care.

We developed and implemented a community-based randomized prenatal care trial in a rural county in eastern China in 2000-2003. The purposes of the trial were to 1) define the optimal content of prenatal care for the context of rural China; 2) to evaluate the effectiveness of such prenatal care on infant and maternal outcomes, using a community-based, well-designed controlled trial in a rural county;3) to describe the process of conducting a controlled study using community resources. The purpose of this paper is to describe the trial implementation and the impact of this community-based trial on the utilization of prenatal care and perinatal outcomes.

### Context of the trial

Carried forward by a wave of economic reforms that began in the late 1970s, the health sector in rural China has experienced major changes in recent decades. Up until the 1980s, the Cooperative Medical Scheme (CMS) had been a collective-based medical care scheme that ensured basic curative and preventive care for most of the rural population. Most of these services were provided free of charge or at a very low cost that was paid by collectives. After the rural collective economy shifted to a market economy in the 1980s, the so-called "household responsibility system" that underpinned the CMS collapsed. That had a major impact on the use and payment of health care, including prenatal care, because farmers had to pay for their health care out of their own pocket [16].

With the economic reforms the responsibility for financing and managing health centers shifted from the county to township authorities [17]. With decentralization, the development of local hospital care plans and the appointment of hospital directors became the responsibility of the township authority. However, the County Health Bureau still transmitted national guidelines and provided technical support when requested [18].

Chinese family planning practices and policy have clearly impacted reproductive health care. In many rural areas, local family planning authorities require married women of childbearing age to have a pregnancy test every 2-3 months. Once a woman is found to be pregnant, the family planning authorities determine whether the pregnancy is according to family planning regulations, or whether the woman is persuaded to abort the baby [19]. In most rural areas, if a couple has a girl, they are allowed to have another child after 4 years, otherwise the one-child family planning policy applies. A pregnancy test gives an early opportunity to provide maternal care education, but it may also lead to women avoiding health care. Some township family planning authorities have required pregnant women to give birth in a designated health facility. The facility was usually the township hospital or township family planning center.

The study was made in a rural county in Anhui province in Eastern China. The area of the county is almost 3000 square kilometers, consisting of mostly flatland. In early 2000, the population was some 900 000, mostly farmers. The GDP (gross national product) ranking of the county is typical of less developed rural areas in China. The healthcare system in the study county consisted of county hospitals and decentralized township- and village-level healthcare services. Specialized obstetric services were available only at the county level. In township hospitals, midwives cared for normal births, although typically there were no systematic prenatal care services. The village-level private clinics were not officially involved in the pregnancy or birth care. All three levels of health care in the county functioned on a fee-for-service basis, and most farmers paid for all health services out-of-pocket [20].

The Chinese household registration system (hukou-system) stipulates where public services-including health care and education-can be obtained [21,22]. Hukou operates differently for agricultural and non-agricultural people, and is also used to regulate rural people's migration to cities. In brief, free or subsidized services are available only in the community one has his or her hukou; births are registered to the hukou community, regardless of where the parents live. Even though there have been several modifications to the system to allow people to have services in the area they live, in the area of our study, the hukou-system resulted in many registered births to women who were no longer living in the area. County health officials estimated that 30-40% of young women had left their hometowns to work elsewhere. However, many come back at the time of delivery and stay for a few months thereafter. Later, children may be left in the care of the grandparents or they move with their parents.

## Materials and methods

The trial was conducted from August 2000 to March 2003; pregnant woman were enrolled in the study up until July 2002. The trial was approved by the ethics committee of the National Research and Development Centre Welfare and Health (STAKES), Helsinki, Finland in January 11, 1999.

For power calculations, perinatal mortality rate (PNMR) was chosen for the indicator. Assuming a 30% reduction in PNMR (estimated 91 per 1000 birth, α = 0.05, and β = 0.1), 1638 births in both groups were needed. Based on the estimated birth rate and population size, we needed 20 townships.

Of the 55 townships in the county, 20 townships were selected that had existing health facilities for the implementation of the trial [20]. The primary criteria for pairing the townships in control and intervention groups were the place of birth (township hospital, township family planning services center and others), fiscal income per capita, average number of prenatal care visits, and the location of the township. Secondary criteria applied were township population, proportion of farmers among the population, assigned birth place (township hospital or township family planning services centre), infant death rate, number of midwives, and number of hospital beds. These data were collected from local government documents and by interviewing local county health officials.

One township was assigned to the intervention and one to control group in each matched pair by the toss of a coin. The randomization was done by the investigators. After the randomization, results were compared once more to ensure the paired townships met the criteria for matching.

### Intervention

Before the trial, we conducted a pilot study in one experimental and one control township to test the feasibility of the data collection methods. In the pilot data were collected from women at different stages of their pregnancy rather than following one group through pregnancy. The intervention trial was not piloted due to difficulties with the local heath authorities in negotiating for a pilot and time constraints.

The intervention consisted of 1) training township midwives, 2) informing women and men in the community of the importance of prenatal care, 3) providing intervention township hospitals with basic medical instruments used in prenatal care (i.e. blood pressure monitors, weighing scales for mothers and newborns, stethoscopes).

#### Training of township midwives

In the intervention group (10 townships), the training of midwives was done in four sessions by a researcher (ZW) and two experienced obstetricians from the county's Maternal and Child Health Care Institute. The first training session lasted two days and later sessions a single day. The midwives were lectured on the content and procedures of prenatal care, as given in the regulation issued by the China Ministry of Health (MOH) in 1989 [14]. The core instruction material used by the trainers was the "Maternal Care Management Approaches for Rural Women", which was issued by the Ministry of Health (MOH) in 1989 [14]. It stipulates that the first prenatal care visit should be made within 13 weeks of gestation, and there should be at least five visits during pregnancy and three after delivery. It specifies that prenatal care should include health education, routine check-ups, laboratory tests, measurements (including height and weight), referral of high risk pregnancy to a high level hospital, and it gives instructions for safe delivery.

During the intervention, the midwives were asked by the researchers to attend meetings organized in the county's maternal and child care institute roughly every three months (eight times) to report and discuss the situation in the prenatal care provision and utilization, including the state of progress and any obstacles encountered. The researchers asked the township midwives to give a standard prenatal care card to each pregnant woman at the first prenatal care visit. The card was for recording the progress of the pregnancy, the use of maternal care services and the pregnancy outcome. The woman was asked to carry the card with her until three postnatal home visits were completed. If a woman discontinued her visits during the course of the pregnancy, the midwives were asked to make home visits.

In addition, the midwives from both the intervention and control groups were asked to attend four one-day training sessions together on keeping medical records and doing women's interviews in hospitals.

#### Informing women and men in the community

The County Health Bureau officials contacted the township's family planning officers and asked them to supply a leaflet on maternal care, entitled "letter to the mother-to-be", to pregnant woman during regular pregnancy testing in the villages. The leaflet was written by the researchers and signed by the County Health Bureau, the Family Planning Committee and the Maternal and Child Health Care Institute. The leaflet advised the pregnant woman to seek prenatal care and to have their delivery in the township hospital. It said that the woman can receive further information about maternity and infant care by consulting midwives in the township hospital. The township midwives facilitated and monitored the distribution of the leaflet.

Secondly, the field research assistant hung posters on the walls of the township hospital, the family planning center, and the village administration offices. The posters were obtained from the provincial Maternal and Child Health Care Institute and consisted of a set of educational posters (12 in total) on daily life during pregnancy (relating to food, nutrition, sleep, work, activities, avoiding poisons etc.) and when and where to have prenatal care and delivery, with emphasis on the importance of a hospital delivery.

#### Trial monitoring

To monitor the trial regularly and to collect data for ongoing evaluations, the researchers hired a medical graduate from the county to work as a field research assistant. She visited every intervention township hospital once a month, monitored the progress of the intervention implementation and gave feedback to the researchers. The researchers made monthly visits to the county to discuss with the County Health Bureau officials and field research assistant any obstacles hindering the trial implementation and to find timely solutions.

### Data collection

Several kinds of data covering the processes and outcomes were collected. Qualitative data included researchers' observations, interviews, group discussions, and field visits by the field assistant and researchers (ZW, YW, KV). Quantitative data included a community-based survey of mothers, a survey conducted by midwives at township hospitals of women giving birth, and routine pregnancy and birth records in township family planning centers.

#### Observations, interviews and discussions

The field research assistant visited the intervention township hospital and their midwives at least once a month. She collected progress data, identified problems and barriers to the intervention, and reported back to the Deputy Director of the County Health Bureau's and also to a researcher (ZW). A researcher (ZW) visited the study county at least every other month, had meetings with the Director and Deputy Director of the Health Bureau and with the field research assistant to discuss progress and to improve the implementation of the intervention. The trial progress was also discussed at routine visits made by the Directors of the County Health Bureau to the intervention townships. The minutes of the meetings and discussions were kept by a researcher (ZW).

The field research assistant and a researcher (ZW) documented the eight workshops attended by midwives, the township hospital manager and county health officials.

The Director and Deputy-Director of the County Health Bureau were (unsystematically) interviewed several times by a researcher (ZW) in regard to the obstacles to and promoters of systematic maternal care. Two researchers (ZW, YW) also interviewed other health administrators, including the Deputy-Director of the county's Mother and Child Health Institute, four directors of the township hospitals (two from the intervention group, two from the control group) and five township midwives (three from the intervention group, two from the control group), again addressing the obstacles to and promoters of maternal care. We also attempted to interview family planning officials, though without success. Each interview lasted between 30 minutes and an hour. Notes were taken during the interview.

#### Hospital based survey

Midwives in the intervention and control townships and in the county level hospitals collected data on women's care, pregnancy and birth, and on the child born, either by asking the mother or extracting from her own medical notes, using a structured data collection form. The information also included the women's home town, thus allowing data to be linked to the study groups. The questionnaire included 49 questions covering parents' background, the mother's reproductive history, infant outcomes, prenatal care and infant health. These data were not used in this paper.

#### Community-based mothers' survey

A survey of mothers was carried out between October 2001 and December 2003.^14 ^This survey covered mothers who had been pregnant during the intervention period and gave birth between April 1, 2001 and March 31, 2003 in the 10 intervention and 10 control townships. Each township in the study county was administratively divided into 6-15 villages. Of the 20 townships, 10 percent were randomly selected to be surveyed in October-December in 2001, with 20 and 30 percent of townships selected in 2002 and 2003, respectively. All mothers who had given birth in the 12 months prior to the survey in these villages were approached for an interview. During that time, 1306 mothers were eligible for the study according to the records kept by the local family planning center. Mothers with dead infants were not approached for interviews.

Interviewers were recruited from among local health workers (not midwives or family planning workers). They were trained to conduct interviews by a researcher (ZW). The structured questionnaire used in the survey included 60 questions covering infant outcomes, infant health and women's knowledge, attitudes, and practices relevant to prenatal, delivery, and postnatal care. The interview was conducted at the mothers' homes. If the mother was not at home at the time of the survey, the father or some other family member responded on her behalf.

Altogether, survey data was collected for 1264 of the 1306 mothers, with 90% of survey respondents being the mother. Data on 42 mothers (3%) were missing: 2 (0.15%) refused, 27 (2.1%) were out of the village; and 13 (1.0%) cases of missing data were for other reasons. There were no differences in the demographic characteristics of the respondents in the intervention and control groups (Table 1). Most (86%) mothers were farmers, and 96% were aged 20-34. A clear majority (63%) were first time mothers, 22% had two children and 14% had three or more children. The intervention group had a larger proportion of higher parity than in the control group.

**Table 1:** Mothers' background by group ^(1)^

	Intervention	Control	Total	P ^(2)^
			% (n)	
(n)	(n = 673) %	(n = 591) %	(n = 1264) %	
Age				
< 20	0.4	0.7	0.6	0.295
20-	73.4	76.1	74.7	
30-	20.8	15.7	18.4	
> = 35	2.4	4.6	3.4	
Missing	3.0	2.9	2.9	
Occupation				
Farmer	80.1	86.8	83.2	0.056
Other	15.2	11.8	13.6	
Missing	4.8	1.4	3.2	
Parity				
1	60.5	66.7	63.4	0.031
2	24.2	20.0	22.2	
3+	15.3	13.4	14.4	
Missing	0.0	0.0	0.0	

(1) Data from community-based survey

(2) P value refers to the difference between the intervention and control group

#### Family planning (FP) routine pregnancy and birth records

The field research assistant abstracted data on pregnancies and their outcomes from the original hand-written work records of the village family planning centers in the 20 study townships. The centers record data on all women who had a positive pregnancy test result. Pregnancy outcome (abortion, including miscarriage, or birth) is also recorded. Due to the hukou-system (regulated residence system) women living and giving birth elsewhere than in the home-community are still marked in the home-community's village records. The records contained data on women's background (birth date, township of residence, and number of living children), pregnancy-related variables (time of last menstrual period, abortion), and infant variables (birth date, gender, plurality, birth order, live birth, stillbirth and early neonatal death). For non-resident women living in the townships, pregnancy data were not usually recorded (information given by local record keepers). Each village family planning center brought their month's records to the administrative family planning office for the purpose of statistics compilation [23].

The outcomes of the pregnancies beginning between August 1, 1999 and July 31, 2002 in the 20 study townships were extracted from the family planning records.

### Data analysis

All interview data and observational notes were reviewed and then analyzed using the framework method [23]. Only the main conclusions from the qualitative data analyses are presented in this paper.

Quantitative data were entered into the computer using EPI-INFO (version 6.04) software and analyzed using SPSS 8.0 for Windows. Since randomization was by community, we adjusted for cluster randomization in the statistical analyses [24]. Differences between the experimental and control groups were tested for with adjusted chi-square tests [25].

The results describing the trial implementation were studied separately for the first and second trial year to see whether the intervention intensified or contamination increased over time. The health outcomes did not differ between the two years, and so combined results are given.

## Results

### Results on trial implementation

#### Local government involvement

This section is based on the researcher's (ZW) and the field research assistant's observations, field notes and surveys of the government officials. The County Health Bureau officials helped to organize the training of township midwives and also arranged eight meetings between township midwives and the researchers. The County Health Bureau officials tried to co-ordinate the relationship between the township hospital (midwives) and the township Family Planning Centers by asking the FP officers at the centers to help supply the information leaflet to mothers-to-be during regular pregnancy testing. This, however, did not work very well because the township Family Planning Center was under the leadership of the County Family Planning Committee rather than the County Health Bureau.

About eight months into the trial (March 2001), the Department of Maternal and Child Health Care at the Provincial Health Bureau decided to carry out a systematic program of maternal care in the whole Anhui province, and assigned this task to the County Health Bureaus. To avoid contamination between the intervention and control groups, we (ZW) tried to persuade the County Health bureau to begin this overlapping initiative in the control townships later than in the other townships. Although the County Health Bureau officials understood the researcher's concern, the maternal care program started only a few months later than in the non-study townships. The managers and midwives of all the study township hospitals were assembled by the County Health Bureau in May 2001 and informed of the provincial government's decision to carry out a province-wide systematic reform of maternal care in May 2001.

Two townships in the intervention group and four in the control group had a prepayment maternal care scheme implemented during our trial, introduced already in the 1990s: every pregnant woman paid 200 RMB (about 24 dollars) in advance to the township hospital, which covered prenatal care, hospital delivery and postnatal care. The scheme promoted the use of maternity care also in the four control townships mentioned above, and thus diluted the trial effects.

#### Training of midwives and their data collection

Analysis of the field notes (ZW) and the hospital-based survey revealed that the training sessions for the midwives in the intervention and control townships were completed as planned. About 95% of available midwives were present in each training session. The 5% of midwives who did not attend were absent because of illness or were not able to leave their work station; they received training on a later day.

#### Informing women in the community

According to the midwives' daily work records, 74% of women who delivered in the hospital in the intervention townships and 71% of the women who delivered in control townships had a prenatal care card.

The distribution of the information leaflet to the pregnant women was not particularly successful (Table 2). According to the discussions between researchers and midwives, in some townships, the FP officers did not welcome the involvement of hospital midwives, thinking that such involvement was beyond the scope of their work and would increase the workload of the FP officers. For midwives, it was also difficult to set their hospital work aside to go with FP officers to visit women in the villages. Another important reason for the failure of this measure was that the midwives lacked motivation since they did not receive subsidies for their extra work.

**Table 2:** Proportions of women receiving information of prenatal care, by group and intervention year (%) ^(1)^

	Intervention	Control	
(n, first year)	(n = 343)	(n = 350)	
(n, second year)	(n = 330)	(n = 241)	P ^(2)^
(n, both years)	(n = 673)	(n = 591)	
Received the study leaflet			
First year	37.0	20.0	0.005
Second year	48.2	44.4	0.748
Both years	42.5	29.9	0.090
Seen the study poster			
First year	27.7	12.6	0.016
Second year	50.6	34.4	0.142
Both years	38.9	21.5	0.014
Received the advice on maternal care from township midwives			
First year	70.0	49.1	0.031
Second year	63.9	64.7	0.942
Both years	67.0	55.5	0.196
Received the advices on maternal care from FP worker			
First year	16.0	17.4	0.797
Second year	33.3	37.8	0.698
Both years	24.5	25.7	0.858

(1) Data from the community base survey

(2) P value refers to the difference between the intervention and control group

Hanging posters (12) relating to prenatal care on the walls of the intervention township hospitals and family planning service centers was carried out as planned. In addition, the field research assistant gave the posters to township midwives to hang on the walls in the village administration office or in the village doctor's office. Based on the researcher assistant's observations, about 70% of villages had the posters in place. According to the community-based survey, about 40% of women in the intervention group saw the study leaflet and poster, more so in the second year of the trial. Some women in the control group had also seen the leaflet and posters, but less often (Table 2).

The proportion of women who received information related to prenatal care from township midwives was higher in the intervention group (70%) than that in control group (49%) in the first year of the trial, though not in the second year (Table 2). The proportion of women who received information related to prenatal care from FP officers did not differ between the two groups.

### Outcome results

#### Maternal care utilization

The community-based survey revealed that differences between the intervention and control groups in the timing of the first prenatal care visit and the frequency of prenatal care visits were small (Table 3). In the intervention group 93% of women gave birth in public hospitals, which was a little higher than in the control group (88%), but the difference was not statistically significant. There was no statistically significant difference in the cesarean section rate between the intervention and control groups (Table 3). Most women (more than 97%) were satisfied with maternal care provided by township midwives, with no difference between the two groups.

**Table 3:** Timing and frequency of prenatal visit and delivery care by group (%) ^(1)^

	Intervention (n = 673)	Control (n = 591)	P^(2)^
1st prenatal visit < = 13 weeks of gestation	40.0	44.5	0.524
No prenatal visit	3.4	3.9	0.758
Prenatal visit > = 5 times	55.9	42.8	0.246
Hospital delivery	92.9	87.5	0.231
C-section	6.5	8.5	0.313

(1) Data from the community base survey

(2) P value refers to the difference between the intervention and control group

There was no difference between the intervention and control groups in the variables measuring women's' knowledge or attitudes towards pregnancy care. 91% of women in the intervention group and 93% in the control group thought that prenatal visits are important and 97% in both groups thought that hospital delivery is important.

##### Content of prenatal care

The community-based survey revealed that only about half of the women in either the intervention or control group had their blood pressure measured at each visit. More women in the control group did not have their blood pressure measured, but the difference between the two groups was not statistically significant. About 43% of the women in both groups had blood tests. There were more women in the control group who had more than one ultrasound scan, but the difference between the two groups was not statistically significant (Table 4).

**Table 4:** Content of prenatal care by group (%)

		Intervention (n = 673)	Control (n = 591)	P value
Blood pressure taken				
	At every visit	49.6	51.3	0.997
	sometimes	38.6	31.1	
	Not at all	8.2	12.5	
Blood test				
	once	34.2	28.9	0.738
	More than once	7.0	12.5	
	no	55.0	53.3	
Ultrasound test				
	1 time	42.8	30.5	0.803
	2 time	13.2	19.0	
	3 and plus	3.3	5.3	
	No	37.0	40.3	
Information on prenatal care				
	Too simple	8.2	11.5	0.065
	Too difficult	2.7	1.9	
	Just OK	52.8	47.9	
	Not sure	20.2	14.9	
	Did not get information	11.9	18.1	
Information on nutrition				
	Needed more	47.1	41.0	0.143
	Needed less	0.2	1.0	
	Just ok	27.3	27.1	
	Not sure	4.9	2.9	
	Did not get information	16.3	23.2	
Information on baby care				
	Needed more	50.1	45.4	0.041
	Needed less	0.2	1.2	
	Just ok	24.2	17.6	
	Not sure	3.3	2.7	
	Did not get information	18.1	28.1	
Information on disease prevention				
	Needed more	49.0	47.0	0.228
	Needed less	0.2	0.3	
	Just ok	30.3	28.4	
	Not sure	4.6	3.1	
	Did not get information	11.7	15.7	

1) Data from the community base survey. Women with no information (varying from 3.7% to 5.8%) are not shown.

2) P value refers to the difference between the intervention and control group

Women would have liked more health promotion information during pregnancy (Table 4). Based on women's reports, the midwives in the intervention group had given somewhat more information on health promotion (Table 4). However, in cases where the information was given, the women considered it equally good in the two groups.

#### Impact of the trial on the pregnancy and perinatal outcomes

There were 1830 recorded pregnancies in the intervention and 1718 in the control group before the intervention period (from August 1, 1999 to July 31, 2000), and 2580 pregnancy records in the intervention and 2550 in the control group during the trial (Table5). In addition, there were 669 women in the intervention and 790 women in the control group who had given birth between January 1, 2001 and December 31, 2002, but with no record of a pregnancy test; these were very likely women who lived elsewhere, but whose deliveries were recorded because of the hukou-system. These women were not included in the analysis

Family planning records showed that the abortion rate, sex ratio at birth and early neonatal death rate (ENDR) both in the intervention and control groups were somewhat lower after the intervention than before it. There were no statistically significant differences between the intervention and control groups in the abortion rate, sex ratio at birth, still birth rate, early neonatal death rate or perinatal mortality (Table 5).

**Table 5:** Pregnancy and perinatal outcomes by group ^(1)^

	Intervention	Control	P ^(2)^
Number of pregnancies			
before intervention	1830	1718	
after intervention	2580	2550	
Abortion rate ( %)			
before intervention	19.1	19.2	0.847
after intervention	17.1	16.8	0.912
number of live births			
before intervention	1423	1362	
after intervention	2094	2062	
Sex ratio at birth			
before intervention	153.0	163.5	0.454
after intervention	144.5	152.8	0.608
Stillbirth rate ( ‰ )			
before intervention	15.5	8.8	0.153
after intervention	7.6	14.1	0.120
Neonatal mortality ( ‰ )			
before intervention	37.9	30.8	0.694
after intervention	37.7	26.2	0.368
Perinatal mortality, ‰			
before intervention	52.6	39.3	0.343
after intervention	42.5	39.3	0.429

1) Data from township family planning records

2) P values are the results of cluster adjusted ordered logistic regression

When we studied the early neonatal mortality (ENDR) by gender, we found that girls' neonatal death rate was much higher than that of boys both in the intervention and control groups. Comparing the time before and after the trial in the intervention group, boys' ENDR dropped from 2.9% to 2.0%, while girls' ENDR increased from 5.2% to 5.7%. In the control group, boys' ENDR increased from 1.3% to 1.8%, while girls' ENDR dropped from 6.1% to 3.6%

## Discussion

This is the first community-based randomized controlled prenatal care trial carried out in rural China. The trial was not fully implemented as planned. Although the training of midwives did occur as planned and some local government support was obtained, the distribution of information on the importance of prenatal care was not particularly successful, partly due to poor cooperation between the township hospital midwives and the family planning officers. In respect of the similar results for health indicators and prenatal care obtained in the intervention and control groups, the likely cause was contamination in the controls and weak implementation of the intervention. There was no impact on still-birth and neonatal death rates or perinatal mortality.

A strength of this study was that we conducted both a process and outcome evaluation concurrently. The main difficulties centered on the rapidly evolving situation in maternity care and also contamination in the control groups.

In the late 1980s, the Ministry of Health issued a regulation requiring local authorities to carry out systematic prenatal care. The regulation was not mandatory and no funding was provided for it. Thus, the provision and use of maternal care varied from place to place. It was up to county or township authorities to decide whether to carry out a systematic prenatal care program. Based on the information of the mid-1990s (at the time when the trial was being planned) we assumed the use of prenatal care to be very low in the study county and township hospitals were under the leadership of county health bureau (including financing and managing township hospitals). However, as shown by our survey, by early 2000 most women in the control townships had already been making several prenatal visits and many were starting care early. And the responsibility for financing and managing township hospitals had shifted from the county to township authorities in late 1990s. This is an example of the very rapidly changing health care in China, reflecting the rapid changes in Chinese society as a whole. The situation should have been mapped more thoroughly and at a time closer to the point of intervention.

The use of prenatal care in the control townships may also be a result of contamination. In the middle of the trial the provincial authorities required the County Health Bureau to carry out county-wide systematic maternal care. That may have resulted in information materials on prenatal care being distributed in the control townships, leading to increased use of prenatal care. This may explain why 56% of mothers in the control group had received advice or information on maternal care from township midwives and 26% from family planning officers. It may also explain why there was a bigger difference between the experimental and control groups in the proportion of women who had received the material in the first year than in the second year.

Another source of contamination may have been contacts between the townships. Since midwives from different township hospitals meet regularly, the midwives from the intervention and control townships may have influenced each other. Furthermore, in the countryside, people often visit relatives and friends in other townships. Some control women may have seen the health education posters while visiting and they may have discussed prenatal care and delivery with relatives or friends they had in intervention townships.

Basic criteria for selecting study townships were that they must have an existing health facility and available hospital staff to support the trial implementation. Therefore, there was little difficulty for township hospitals to carry out prenatal care and delivery services. On the other hand, the criteria may have worked to select the best townships, which of itself could have yielded an increased proportion of women using prenatal care, irrespective of our intervention.

Even though the county officials were supportive, they did not have full control over the townships. Health care administration was partly decentralized from the county to the township level, with township hospitals partially funded by township authorities. The County Health Bureau supervised the townships and their professionals, but they could not appoint staff or regulate their work assignments.

In the study county, the family planning (FP) sector has more public resources than the health sector. The township's FP officers were under the leadership of the County Family Planning Committee and township authorities. So the County Health Bureau officials were not able to coordinate the work of the FP officers with the township midwives. This could explain why one of our intervention procedures, the dissemination of leaflets on maternal care to future mothers during pregnancy testing, was not successfully implemented. We were not given permission to discuss the matter with family planning officials at the county or higher level so as to remedy the situation.

In addition, the FP officers were not paid for supplying the leaflets and it was not part of their ordinary work. So they lacked enthusiasm to cooperate with the health sector. Furthermore, there were only one or two midwives engaged in the trial work in each township hospital, and they were often too busy to leave the hospital, which decreased their participation in the information dissemination. Unfortunately, we did not collect data on how many leaflets were actually supplied to pregnant women.

To profit from fees-for-services, township hospitals have to be of high quality and acceptable to the patients. The midwife training was consistent with the interests of hospitals to remain competitive and it was not unexpected that the training sessions were carried out smoothly.

Some of our indicators turned out to be unsuitable for measuring the impacts of the trial intervention. Our study has shown that the early neonatal death rate is much higher among girls than boys, both before [19,20] and after the trial. If the impact of the family planning policy is larger on perinatal mortality than maternal care, then it is hard for any health care intervention to have an effect on perinatal health outcomes. Typically, in large-scale cluster randomization trials that evaluate public health interventions at community level, the expected effects on morbidity and mortality tend to be modest, although still of public health relevance [26]. A second unsuitable indicator was the hospital delivery rate. It was already high, and in such a situation it is hard to distinguish a difference between study groups.

We could not find any other studies that were sufficiently similar to allow a comparison with our trial. If such was the case, it would have been useful to compare the factors affecting success or failure in introducing a community-based intervention aimed at increasing the utilization of prenatal care.

## Conclusions

Our study showed that a community based randomized controlled trial could not be fully carried out in rural China as planned due to the changing political landscape, the complexity of the socio-economic situation and a lengthy planning stage, The trial was adversely affected by policy changes at a higher level of government, the decentralization of the decision-making in health care, and the poor cooperation between family planning and health sectors at township level. This kind of cooperation is crucial to the success any intervention in maternity care in rural China, given that the family planning sector is better financed than the health sector in townships. This trial could be well planned but was hard to implement. Thus, it was not able to answer if prenatal care is able to improve perinatal outcomes.

## Competing interests

The authors declare that they have no competing interests.

## Authors' contributions

ZW was involved in the conception and design of the research idea, coordination and acquisition of the data, analyses and interpretation of the data, and in drafting, revising the manuscript. KV was involved in the conception and design of the research idea, analyses and interpretation of the data, and in drafting, revising the final manuscript; YW was involved in the coordination and acquisition of the data, analyses and interpretation of the data, and in revising the manuscript. EH was involved in the conception and design of the research idea, analyses and interpretation of the data, and in drafting, reviewing and revising the manuscript.

## Ethical approval

The study was approved by the STAKES ethics committee, 11 January, 1999

## Pre-publication history

The pre-publication history for this paper can be accessed here:

http://www.biomedcentral.com/1472-6963/11/92/prepub

## References

[B1] Drazancic A Antenatal care in developing countries. What shoud be done? Journal of Perinatal Medicine 2001 29 3 188 98 10.1515/JPM.2001.028 11447923

[B2] Lumbiganon P Winiyakul N Chongsomchai C Chaisiri K From research to practice: the example of antenatal care in Thailand Bulletin of World Health Organization 2004 82 10 746 9 PMC2623043 15643795

[B3] Zanconato G Msolomba R Guarenti L Franchi M Antenatal care in developing countries: the need for a tailored model Seminars in Fetal and neonatal Medicine 2006 11 1 15 20 10.1016/j.siny.2005.10.002 16364704

[B4] Hong R Ruiz-Beltran Martin Impact of prenatal care on infant survival in Bangladesh Maternal and Child Health Journal 2007 11 2 199 206 10.1007/s10995-006-0147-2 17136460

[B5] Khan-Neelofur D Gulmezoglu M Villar J WHO antenatal care trial group Who should provide antenatal care for low-risk women, and how often? A systematic review of randomized controlled trials Paediatric and Perinatal Epidemiology 1998 12 suppl 2 7 26 9805721 10.1046/j.1365-3016.12.s2.6.x

[B6] White DE Fraser-Lee NJ Tough S Newburn-Cook CV The content of prenatal care and its relationship to preterm birth in Alberta, Canada Health care for Women Internatational 2006 27 9 777 92 10.1080/07399330600880335 17060178

[B7] Lu MC Tache V Alexander GR Kotelchuck M Halfon N Preventing low birth weight: is prenatal care the answer? Journal of maternal-fetal and neonatal Medicine 2003 13 6 362 80 12962261 10.1080/jmf.13.6.362.380

[B8] Ickovics JR Trace S Westdahl C Group prenatal care and perinatal outcomes: A randomized controlled trial Obstetrics and Gynecology 2007 110 2, part1 330 339 10.1097/01.AOG.0000275284.24298.23 17666608 PMC2276878

[B9] Carroli G Rooney C Villar J How effective is antenatal care in preventing maternal mortality and serious morbidity? An overview of the evidence Paediatric and perinatal Epidemiology 2001 15 suppl 1 1 42 11243499 10.1046/j.1365-3016.2001.0150s1001.x

[B10] Munjanja S Lindmark G Nystrom L Randomized controlled trial of a reduced-visits program of antenatal care in Harare, Zimbabwe The Lancet 1996 348 9024 364 66 10.1016/S0140-6736(96)01250-0 8709734

[B11] Vidaff AC Franzini L Low M The unrealized potential of prenatal care. A population health approach Journal of Reproductive Medicine 2003 48 11 837 42 14699994

[B12] Rumbold AR Cunningham J A review of the impact of antenatal care for Australian indigenous women and attempt to strengthen these services Maternal and Child Health Journal 2008 12 1 83 100 10.1007/s10995-007-0216-1 17577650

[B13] Greg R Alexander MK Assessing the role and effectiveness of prenatal care: History, challenges, and directions for future research Public Health Reports 2001 116 306 316 12037259 10.1016/S0033-3549(04)50052-3 PMC1497343

[B14] Wu Z Viisainen K Li X Hemminki E Maternal care in rural China: a case study from Anhui province BMC Health Services Research 2008 8 55 10.1186/1472-6963-8-55 18331626 PMC2329627

[B15] Wu Z Li X Gao J Xu L Maternal care use in China from 1993to 2003 Chinese Primary Health Care 2005 19 9 45 47

[B16] Fang J Health sector reform and reproductive health services in poor rural China Health Policy and Planning 2004 19 Suppl.1 140 49 10.1093/heapol/czh044 15452014

[B17] Bloom G Tang S Rural health prepayment scheme in China: toward a more active role for government Social Science and Medicine 1999 48 7 951 60 10.1016/S0277-9536(98)00395-5 10192561

[B18] Tang S Bloom G Decentralizing rural health services: a case study in China International Journal of Health Planning and Management 2000 15 3 189 200 10.1002/1099-1751(200007/09)15:3<189::AID-HPM590>3.0.CO;2-Q 11184653

[B19] Wu Z Viisainen K Hemminki E Determinants of high sex ratio among newborns: A cohort study from rural Anhui province, China Reproductive Health Matters 2006 14 27 172 80 10.1016/S0968-8080(06)27222-7 16713892

[B20] Wu Z Viisainen K Wang Y Hemminki E Perinatal mortality in rural China: restrospective cohort study BMJ 2003 327 319 22 10.1136/bmj.327.7427.1319 PMC286318 14656839

[B21] Wu X Treiman DJ The household registration system and social stratification in China:1955-1996 Demography 2004 41 2 363 84 10.1353/dem.2004.0010 15209045

[B22] Wong L Wai-po H Reforming the household system: a preliminary glimpse of the blue chop household registration system in Shanghai and Shenzhen International Migration Review 1998 32 4 974 94 10.2307/2547668 12294304

[B23] Ritchie J Spencer L Qualitative data analysis for applied policy research Analyzing Qualitative Data Routledge, London 173 194 (Bryman A. & Burgess R.G. etc.)

[B24] Donner A Brikett N Buck C Randomisation by cluster: sample size requirements and analysis Am J Epidemiology 1981 114 906 14 10.1093/oxfordjournals.aje.a113261 7315838

[B25] Donner A Donald A The statistical analysis of multiple binary measurements Journal of Chronic Diseases 1988 41 899 10.1016/0895-4356(88)90107-2 3183697

[B26] Susser M The tribulations of trials--Intervention in communities [Editorial] American Journal of Public Health 1995 85 2 156 158 10.2105/AJPH.85.2.156 7856769 PMC1615322

